# Novel iflavirus species discovered through virome analysis of cotton field-collected aphids

**DOI:** 10.1186/s12864-026-13132-9

**Published:** 2026-07-10

**Authors:** Chaoyang Zhao, Isabella Owens, Alana L. Jacobson, Cesar Escalante, Raman Bansal, Kathleen M. Martin, Kipling S. Balkcom

**Affiliations:** 1 National Soil Dynamics Laboratory, USDA-ARS, Auburn, AL USA; 2 Department of Entomology and Plant Pathology, Auburn University, Auburn, AL USA; 3 San Joaquin Valley Agricultural Sciences Center, USDA-ARS, Parlier, CA USA; 4 Department of Botany & Plant Pathology, Purdue University, West Lafayette, IN USA

**Keywords:** Complete genome sequencing, Insect virus discovery, Plant-mediated transmission, RNA virus diversity, Virus evolution

## Abstract

**Background:**

Insect-associated viruses (viromes) shape insect biology and agroecosystems, yet aphid viromes remain undercharacterized. The cotton aphid, *Aphis gossypii*, is a globally distributed pest with a broad plant host range and demonstrated virus-vector competence, making it well suited for investigating virome composition at the plant–insect–virus interface. In this study, we profiled the virome of field-collected aphid alates, which were dominated by *A. gossypii* but included additional aphid species in some samples, across 15 cotton fields in Alabama, USA, to assess taxonomic diversity, relative viral abundance, and focal aphid-associated viruses.

**Results:**

Metatranscriptomic sequencing of aphid alates revealed differences in taxonomic composition among county-level libraries, including viruses and diverse prokaryotic and eukaryotic taxa. Fifty-eight viral contigs > 1 kb were assembled, of which 20 were assigned to seven families: *Dicistroviridae*, *Iflaviridae*, *Mitoviridae*, *Nudiviridae*, *Partitiviridae*, *Phasmaviridae*, and *Solemoviridae*. Representative contigs from six families (all except *Phasmaviridae*) and five additional family-unassigned viruses were validated by PCR and Sanger sequencing. Three iflaviruses (RrIV, AgIV1, and AgIV2), positive-sense single-stranded RNA viruses, were discovered, including one associated with *Rhopalosiphum rufiabdominale* and two with *A. gossypii*. Their complete genomes were determined using PCR-based resequencing and 5′/3′ RACE, each comprising a single open reading frame that encodes a polyprotein. Sequence identity and phylogenetic analyses indicate that the newly identified viruses are putative new species and form a distinct clade together with previously reported aphid iflaviruses within *Iflaviridae*. AgIV1 and AgIV2, despite being associated with the same host species, were not monophyletic within this clade, consistent with cross-species transmission among aphid hosts. Strand-specific RT-PCR detected negative-strand RNA for AgIV1 and AgIV2, suggesting replication in aphids. Given their low field prevalence, we propose the names *Iflavirus furtiva* (RrIV), *Iflavirus obscurata* (AgIV1), and *Iflavirus rarivira* (AgIV2).

**Conclusions:**

The aphid virome is taxonomically diverse and shows county-level differences in relative viral contig abundance. We identified seven viral families and three complete iflavirus genomes, providing a foundation for further investigating their host range, transmission, and potential impacts on aphid biology and cotton production.

**Supplementary Information:**

The online version contains supplementary material available at https://doi.org/10.1186/s12864-026-13132-9.

## Introduction

 Insects harbor diverse microbial communities that coevolve with their hosts through complex, dynamic interactions [1]. These communities include bacteria, fungi, protists, and viruses, each contributing uniquely to host biology. While some microbes act as insect pathogens, others provide benefits such as nutrient provisioning, immune modulation, and protection against infection [2, 3]. Among these constituents, viruses (the virome) remain comparatively understudied. Metagenomic and metatranscriptomic surveys continue to uncover extensive viral diversity in insects, yet the ecological roles, transmission routes, and evolutionary dynamics of most lineages are still unclear [4]. Clarifying virome composition and function is therefore important for understanding insect physiology and evolution, as well as predicting ecosystem and agricultural impacts [4, 5].

In herbivorous insects, viral associations can strongly influence plant health and crop productivity [6]. Many insect species are vectors of plant viruses, facilitating spread among host plants within and across fields. Beyond passive carriage, vector-mediated mechanisms can increase viral dissemination [7, 8], and host-plant infection can enhance insect performance and feeding efficiency [9]. These reciprocal effects underscore the need to study insect viromes within a tripartite plant–insect–virus framework, in which feedback among partners shapes ecological and evolutionary outcomes.

The cotton aphid, *Aphis gossypii*, is a globally important pest with a broad host range [10, 11]. In Alabama, it is the most abundant aphid species in cotton fields during the growing season, with populations typically peaking from late June to mid-July [12, 13]. *A. gossypii* transmits numerous plant viruses and is also the only confirmed insect vector of cotton leafroll dwarf virus (CLRDV), a pathogen associated with severe yield losses in South America and now present in the United States [13–17]. This combination of polyphagy, high dispersal, and demonstrated vector competence makes *A. gossypii* a compelling system for dissecting how virome composition relates to aphid ecology and plant disease risk [12–14]. Yet, despite its agricultural importance, the virome of *A. gossypii* remains poorly characterized. Previous research has emphasized bacterial symbionts and their effects on host traits [18–20], leaving open fundamental questions about the diversity, distribution, and functional significance of aphid-associated viruses. Moreover, studies in other insects indicate that geographic variation—shaped by host genetics, local environments, and plant communities—can structure insect viromes [21, 22]. Whether similar spatial patterning occurs in *A. gossypii* remains unknown.

In this study, we address these gaps by profiling the virome of cotton field-collected aphids across five cotton-growing counties in Alabama, USA, using high-throughput RNA sequencing (RNA-seq) and a standardized bioinformatics workflow. Although collections were dominated by *A. gossypii*, molecular analyses revealed the presence of additional aphid species in a subset of samples. We catalogued the diversity of viruses associated with field populations, including taxa lacking confident family-level assignments; described differences in relative viral abundance among county-level libraries; and validated representative viruses by PCR and Sanger sequencing. We then focused on *Iflaviridae*, reporting three aphid-associated iflaviruses for which we assembled complete genomes, conducted phylogenetic analyses, and performed strand-specific RT-PCR to assess replication-consistent transcripts in aphids. Together, these analyses characterize the diversity of the aphid-associated virome and provide resources to probe virus origins, host range, transmission routes, and functional impacts in cotton ecosystems.

## Materials and methods

### Aphid collection

Alate aphids (Hemiptera: Aphididae) were collected from cotton fields in five Alabama counties (Autauga, Escambia, Henry, Lee, and Macon) between June 29, 2024, and July 5, 2024. In each county, three fields were sampled (Table S1). Aphids were transported on ice and processed in the laboratory. For each plant, all live alates were pooled into a single 1.5-mL microcentrifuge tube, placed in 100% ethanol, and immediately stored at − 80 °C until RNA isolation. Pooled alates from the same plant were considered one sample, and ten pooled samples were collected per field. 

### RNA extraction and sequencing

Total RNA was extracted from plant-level samples (pooled alates from a single plant) using the RNeasy Mini Kit (QIAGEN, Hilden, Germany). For each county, RNA from 30 plant-level samples was combined by adding 6 µL from each sample (180 µL total) and concentrated by ethanol precipitation [23]. The resulting county-level RNA pool was treated with TURBO DNase (Invitrogen, Carlsbad, CA, USA) to remove residual genomic DNA.

Five county-level RNA pools (AgA, AgE, AgH, AgL, and AgM; Table S1), each representing one county, were submitted to Novogene Corp. Inc. (Sacramento, CA, USA) for sequencing. Ribosomal RNA was depleted using the Ribo-Zero rRNA Removal Kit (Illumina, CA, USA). Libraries were prepared according to the manufacturer’s protocol (fragmentation, reverse transcription, PCR amplification), and sequenced on an Illumina NovaSeq 6000 to generate 150 bp paired-end reads. 

### RNA-seq analysis and viral contig prediction

RNA-seq raw reads were filtered to remove adapters and low-quality reads, including reads with > 10% ambiguous nucleotides or > 50% bases with Phred < 5. To identify the aphid species present in each RNA pool, clean reads from five county-level libraries were separately de-novo assembled using Trinity (v2.15.1) [24]. The *A. gossypii* COI barcoding sequence (NCBI accession EU701377.1) was used to search against each of the five Trinity assemblies with BLASTn (E-value < 1e-10). All hit sequences were subsequently queried against the NCBI core nucleotide database (accessed April 06, 2026), and those for which aphid COI sequences were the top BLAST hits were retained for species identification. These sequences were examined for the presence of the complete 658-bp barcode region [25]. The retrieved 658-bp sequences were searched against the BOLD database (Animal species-level library, Public + Private; operating mode: Genus and Species Search; https://www.boldsystems.org/, accessed April 06, 2026) [26] for final species identification.

For taxonomic analysis of the RNA-seq reads, clean reads from each of the five libraries were first mapped to the *A. gossypii* reference genome (NCBI accession GCA_020184175.2) using Bowtie2 (default settings) [27]. Unmapped reads were designated as non-host and taxonomically classified with Kraken2 [28].

Viral contig assembly and taxonomic analysis followed a SPAdes + DIAMOND/MEGAN workflow [29]. Clean non-host reads from the five libraries were concatenated to assemble a metatranscriptome with SPAdes (v3.15.5) in meta mode [30]. Assembled contigs were searched against the NCBI non-redundant (NR) protein database (downloaded August 28, 2023) using the DIAMOND BLASTx (v2.1.8) function. DIAMOND alignment archive (DAA) files were processed with MEGAN (v6_25_3) using the mapping database ‘megan-map-Feb2022.db’ in long-read mode via MEGANIZER. Contigs were analyzed in MEGAN’s interactive mode, and those classified as viral and ≥ 1,000 nt were retained for downstream analyses.

### Viral contig annotation and relative abundance analysis

Putative viral contigs were queried against NR at the NCBI BLAST web portal with BLASTx (E-value < 1e-5; accessed February 06, 2025), restricting hits to ‘Viruses’ (taxid 10239). For each contig, we recorded the top hit (based on highest bit‑score and lowest E‑value), taxonomic assignment, and percentage identity.

To estimate relative abundance, total clean RNA-seq reads (including aphid reads) from each county were independently aligned to the viral contigs, and alignments were filtered at mapping quality ≥ 30 using SAMtools (v1.17) [31]. Read counts were normalized as RPKM (reads per kilobase per million total reads). Because viral abundance values spanned several orders of magnitude, RPKM values were transformed as log2(RPKM + 1) and then converted to z-scores (mean = 0, SD = 1, range − 2 to 2) in R (v4.3.2), representing a row-wise normalization to visualize relative abundance patterns of each contig across counties. Heatmaps were generated using a five-stop blue-white-red color gradient with ComplexHeatmap (v2.18.0) [32]. Annotations included viral family (categorical) and BLAST percent identity (continuous).

### Validation of viral contigs

Virus validation was conducted using the QIAGEN One-Step RT-PCR Kit according to the manufacturer’s instructions. Primer sequences are listed in Table S2-A. Aphid RNA quality was confirmed using primers targeting *A. gossypii elongation factor 1-alpha* (*EF1α*; NCBI accession XM_050204362.1). Amplicons were cloned with the TOPO TA Cloning Kit (Invitrogen) and confirmed by Sanger sequencing of plasmid inserts. 

### Phylogenetic analysis

For the two contigs (978 and 2373) putatively derived from *Partitiviridae*, we analyzed their RNA-dependent RNA polymerase (RdRp) amino acid sequences alongside representatives from all five recognized genera of *Partitiviridae* and sequences reported in a recent cotton virome study (https://github.com/ceesgua91/Cotton_virome) [33].

A separate phylogenetic analysis was conducted to resolve relationships among the identified iflaviruses. For brevity, we use the common names Rhopalosiphum rufiabdominale iflavirus (RrIV), Aphis gossypii iflavirus 1 (AgIV1) and Aphis gossypii iflavirus 2 (AgIV2) to refer to the viruses represented by contigs 32, 33, and 37, respectively; contig 8504 corresponds to a truncated fragment. We analyzed polyprotein sequences from three species identified in this study (RrIV, AgIV1, and AgIV2), six aphid iflaviruses characterized by Rozo-Lopez et al. [34], and all ICTV-reconciled iflavirus species.

Protein sequences were aligned with MAFFT (v7.505). Poorly aligned regions were removed with trimAl (v1.2) based on a gap threshold of 0.25. Bayesian phylogenies were inferred in MrBayes (v3.2.7) using the mixed amino acid model. Two independent runs of four chains were executed until the average standard deviation of split frequencies was < 0.05. The first 25% of samples were discarded as burn-in, and 50% majority-rule consensus trees were computed.

### PCR-based resequencing and 5’/3’ terminal RACE of aphid iflavirus genomes

Three nearly complete iflavirus genomes were assembled from RNA-seq reads. Overlapping PCR primers (Table S2-B) were designed to generate 1.0-2.2 kb amplicons that span each genome to confirm the assemblies. Genome termini were obtained by 5′/3′ rapid amplification of cDNA ends (RACE) using the Invitrogen 5′ and 3′ RACE System following the manufacturer’s protocol. Specifically, complete genome sequences of RrIV and AgIV2 were obtained from pooled Macon RNA samples, and the complete genome of AgIV1 was obtained from pooled Escambia RNA samples using overlapping RT-PCR amplification and 5’/3’ RACE. Gene-specific and adaptor primers are listed in Table S2-C. All PCR and RACE amplicons were cloned and Sanger-sequenced from plasmids.

### Pooled screening, individual detection, and host association analysis of aphid iflaviruses

Each county‑level RNA pool was first screened for the three iflaviruses (RrIV, AgIV1, and AgIV2) using QIAGEN One-Step RT-PCR with virus‑specific primer pairs (Table S2-D). Pools testing positive were deconvoluted by testing individual RNA samples (*n* = 30 per pool). Positive individual samples were subsequently used to test their host association by amplification, cloning, and sequencing of COI barcode fragments following the previously described protocol [35].

### Detection of negative-strand RNA in aphid iflaviruses

Virus replication was assessed following Valles et al. [36] using adaptor-tagged, strand-specific RT-PCR. Briefly, cDNA specific to the negative (replicative) strand was synthesized with an adaptor-tagged gene-specific primer (Table S2-E). As a control, cDNA specific to the positive-strand RNA was synthesized with an untagged gene-specific primer (Table S2-E). Unincorporated single-stranded DNA was digested with Exonuclease I. PCR conditions were: 94 °C for 2 min; 35 cycles at 94 °C for 15 s, 56 °C for 20 s, and 72 °C for 40 s; final extension 72 °C for 5 min. Each assay (negative-strand test and positive-strand control) included corresponding no-template controls. PCR products were analyzed by gel electrophoresis.

## Results

### Aphid species composition and metatranscriptomic profiles across five Alabama counties

**Fig. 1:**
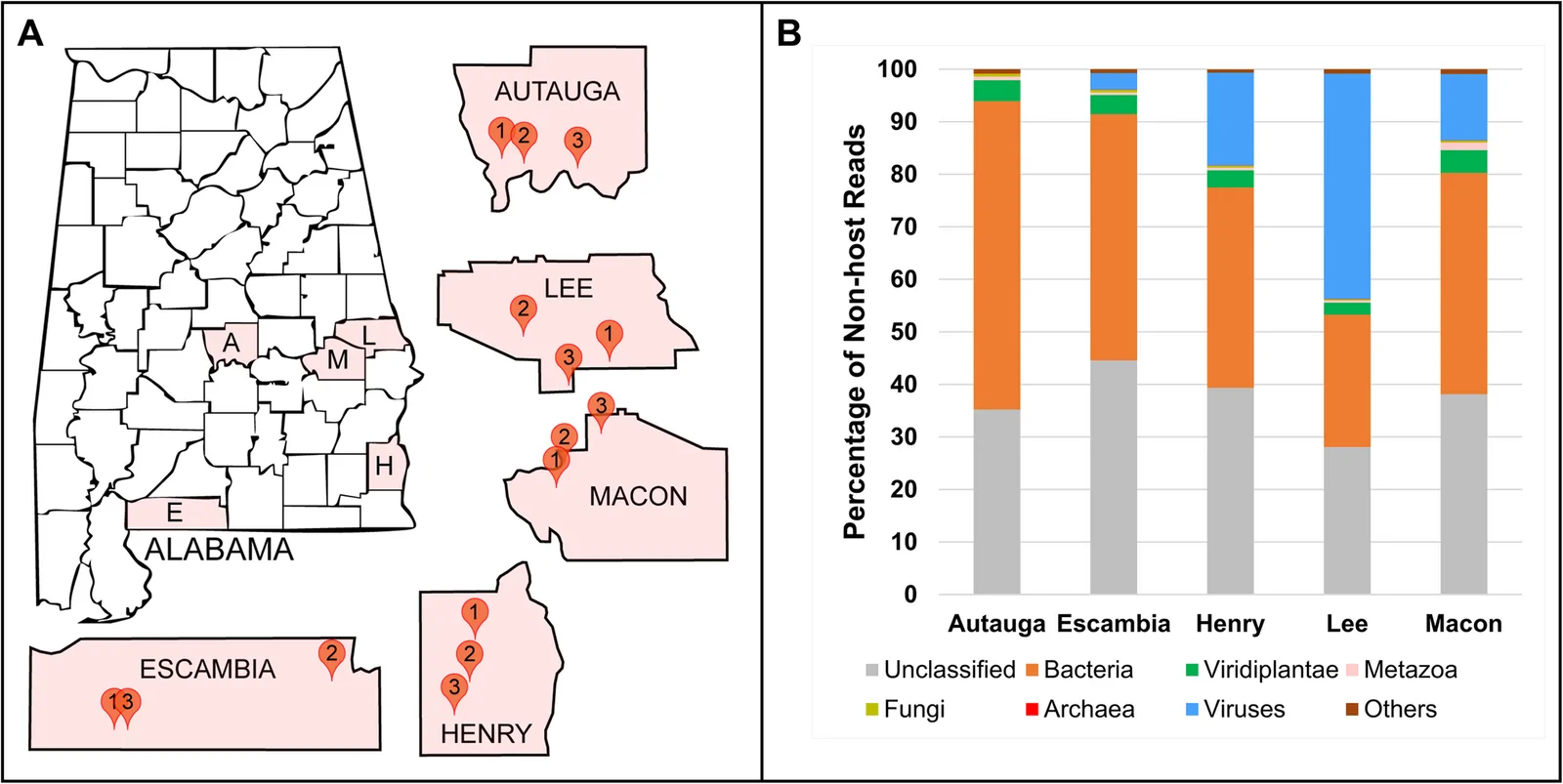
Geographic sampling and metatranscriptomic profiling of cotton field-collected aphids.** A** Sampling locations in five Alabama counties —Autauga (A), Escambia (E), Henry (H), Lee (L), and Macon (M) —with aphids collected from three cotton fields per county. **B** Taxonomic composition of non-host RNA-seq reads per county, classified with Kraken2

RNA sequencing was conducted on five aphid libraries, each comprising pooled RNA from alates collected from 30 cotton plants across three field sites within one Alabama county (one county per library) (Fig. 1A; Table S1). Raw read counts per sample ranged from 18.1 to 25.6 million, yielding 17.6 to 24.4 million high-quality (clean) reads (Table S3). GC content was consistent across samples (35.8%-37.2%). Reads unmapped to the *A. gossypii* genome, designated as non-host reads, ranged from 5.7 to 10.4 million, accounting for 28.9% − 45.6% of the total clean reads (Table S3).

Because not every individual aphid was morphologically identified prior to RNA extraction, the pooled RNA samples may contain mixtures of aphid species. To determine the species composition in each county-level RNA sample, we used de novo Trinity assemblies to recover the 658-bp COI barcode sequences. These COI sequences were queried against the BOLD Identification System, which showed that three counties (Autauga, Escambia, and Henry) contained only a single species, *A. gossypii*. In contrast, two aphid species (*A. gossypii* and *Rhopalosiphum rufiabdominale*) were detected in the Lee County sample, while three species (*A. gossypii*, *R. rufiabdominale*, and *Melanaphis sorghi*) were present in the Macon County sample (Table S4).

Taxonomic classification of non-host reads using Kraken2 revealed differences in taxonomic composition among the five Alabama counties (Fig. 1B). Bacterial reads dominated in Autauga (58.7%), Escambia (46.9%) and Macon (42.1%), but were comparatively lower in Henry (38.1%) and Lee (25.2%). Conversely, viral reads were highly enriched in Lee (42.9%) and Henry (17.7%), but were scarcely represented in Autauga (0.03%) and Escambia (3.13%). Minor proportions of reads were assigned to “Viridiplantae”, “Metazoa”, and “Fungi” (< 5%), while “Archaea” and “Others” remained consistently below 1%. Notably, unclassified reads constituted a substantial portion across all counties, ranging from 28.1% to 44.6%.

### Characterization, relative abundance, and validation of the aphid virome

**Fig. 2:**
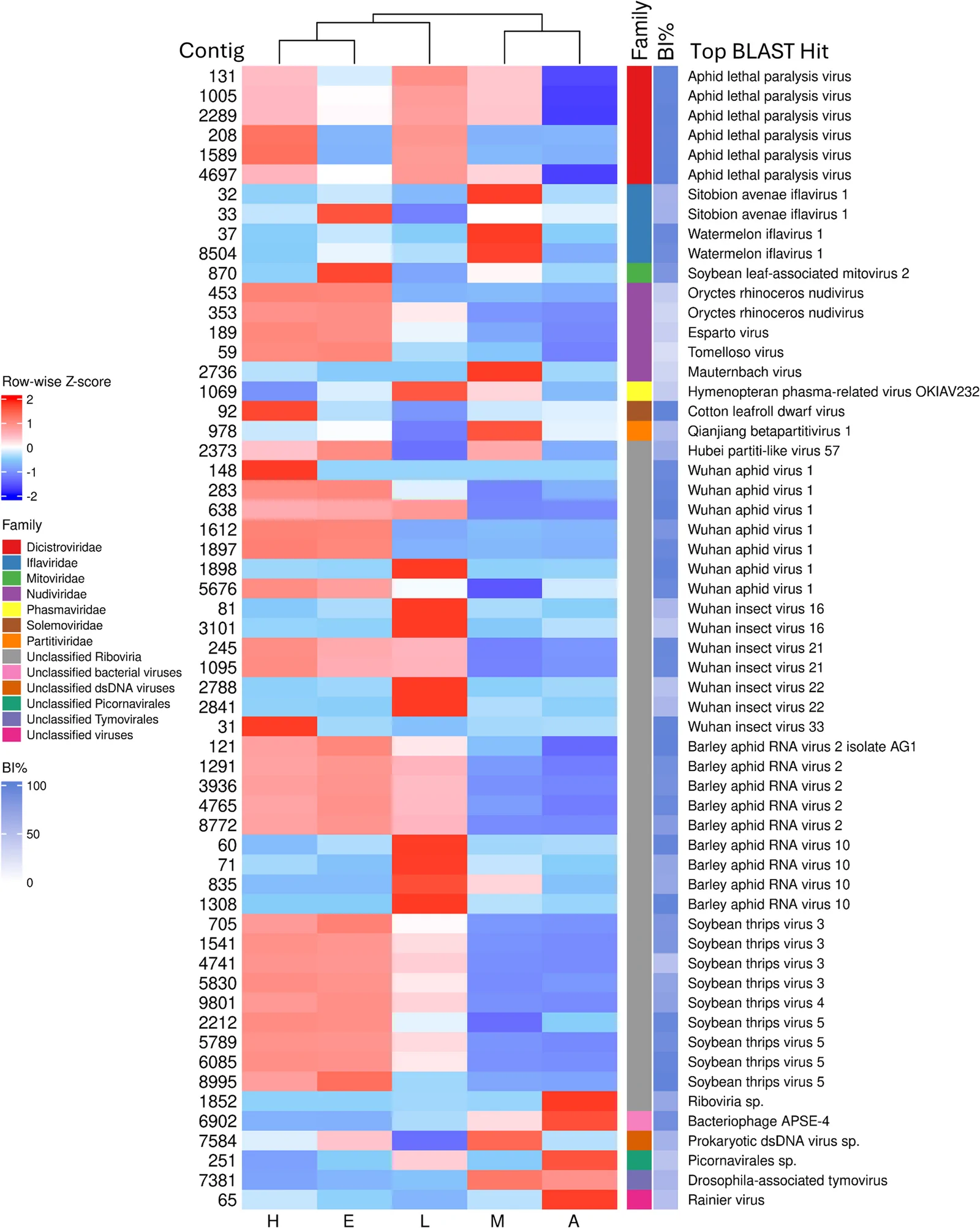
Heat map and taxonomic annotation of putative viral contigs. Relative abundance of viral contigs is shown for field-collected aphid samples from five Alabama counties: Autauga (A), Escambia (E), Henry (H), Lee (L), and Macon (M). Values represent log2-transformed RPKM (log2[RPKM + 1]) that were row-wise normalized to z-scores (mean = 0, SD = 1), indicating relative abundance patterns of each contig across counties. Contigs (≥ 1,000 nt) were annotated based on their top BLAST hit in the NCBI NR database, including species name, BLAST percent identity (BI%), and viral family

The metatranscriptome assembled from aphid samples uncovered a diverse and complex viral community. Taxonomic analysis identified 126 viral contigs, of which 58 exceeded 1 kb in length. BLASTx-based homology searches assigned 19 contigs (> 1 kb each) to seven viral families: *Dicistroviridae* (6 contigs), *Nudiviridae* (5), *Iflaviridae* (4), *Mitoviridae* (1), *Phasmaviridae* (1), *Solemoviridae* (1), and *Partitiviridae* (1) (Fig. 2; Table S5). Among the remaining 39 contigs lacking confident family-level assignments, many showed high sequence similarity to previously reported insect-associated viruses, including Wuhan aphid virus 1 (WAV1; 7 contigs), WAV16 (2), WAV21 (2), WAV22 (2), WAV33 (1), barley aphid RNA virus 2 (WARV2; 5), WARV10 (4), soybean thrips virus 3 (STV3; 4), STV4 (1), and STV5 (4) (Fig. 2; Table S5). Viruses exhibited varied relative abundance patterns across county-level libraries at the time of aphid collection (Fig. 2). Because libraries were generated from pooled samples, these values should not be interpreted as measures of prevalence or geographic distribution.

**Fig. 3:**
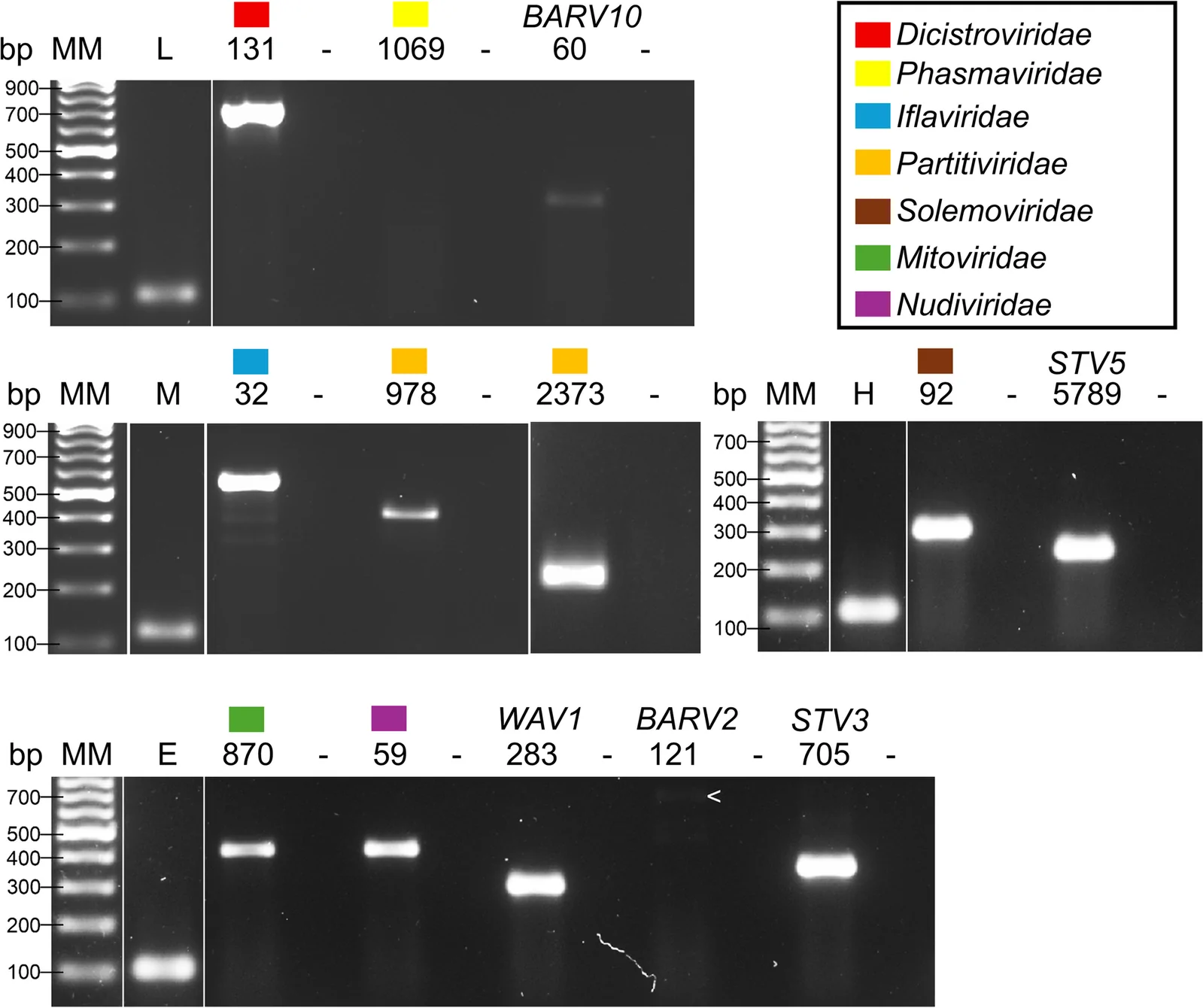
PCR validation of selected viral contigs. RT-PCR products confirm the presence of contigs detected in aphid RNA pools from Lee (L), Macon (M), Henry (H), and Escambia (E). Family-assigned contigs are color-coded by viral family in the panel header; contigs without confident family assignment are labeled by their top BLASTx hits (barley aphid RNA virus 2, BARV2; barley aphid RNA virus 10, BARV10; soybean thrips virus 3, STV3; soybean thrips virus 5, STV5; Wuhan aphid virus 1, WAV1). Minus (-) lanes are no-template controls. The white arrow in the E RNA pool marks a faint specific band for contig 121. RNA integrity/RT competence was verified by amplification of the *Aphis gossypii* housekeeping gene *Elongation Factor 1-α* (*EF1α*); the *EF1α* product is shown adjacent to the molecular marker (MM)

A single contig (978) was initially identified with highest sequence similarity to Qianjiang betapartitivirus 1, a member of the *Partitiviridae* family (Fig. 2). Concurrently, contig 2373 aligned most closely with Hubei partiti-like virus 57, suggesting a potential *Partitiviridae* affiliation, although its family-level classification remained unresolved. PCR and Sanger sequencing confirmed the presence of contig 2373 (Fig. 3). Phylogenetic analysis, incorporating representative species from all five recognized *Partitiviridae* genera (Table S6), placed contig 978 within the *Betapartitivirus* genus and contig 2373 within *Deltapartitivirus*, with strong support (posterior probability = 100% and 94%, respectively) (Fig. S1).

PCR and Sanger sequencing confirmed the presence of six of the seven identified viral families: *Dicistroviridae* (contig 131), *Iflaviridae* (contig 32), *Partitiviridae* (contig 978), *Solemoviridae* (contig 92), *Mitoviridae* (contig 870), and *Nudiviridae* (contig 59) (Fig. 3). *Phasmaviridae* (contig 1069) was not detected by PCR. Additionally, five viruses (BARV2, BARV10, STV3, STV5, and WAV1), each represented by ≥ 3 contigs but lacking definitive family-level assignments, were PCR-validated (Fig. 3). 

### Novel aphid *Iflaviridae* species

Four contigs (32, 33, 37, and 8504) classified within the *Iflaviridae* family were identified in the aphid virome (Fig. 2; Table S5). BLASTx searches revealed that contigs 32 and 33 shared highest sequence similarity with Sitobion avenae iflavirus 1 (SaIV1), whereas contigs 37 and 8504 were most similar to watermelon iflavirus 1 (WIV1). Notably, contigs 32, 37, and 8504 showed relatively higher RNA-seq read abundance in Macon County compared to the other four counties. In contrast, contig 33 showed relatively higher abundance in Escambia County (Fig. 2). These patterns suggest the presence of multiple *Iflaviridae* species or strains within the aphid virome.

Contigs 32, 33, and 37 represent near-complete genomes (10,190 nt, 10,120 nt, and 9,384 nt, respectively), consistent with the typical *Iflaviridae* genome size of approximately 9–11 kb (https://ictv.global/report/chapter/iflaviridae/iflaviridae). Based on their genome length and sequence divergence, we hypothesized that these contigs correspond to three distinct *Iflaviridae* species. Contig 8504, however, is only 1,071 nt in length and likely represents a truncated genomic fragment (Table S5).

To evaluate whether contigs 32, 33, and 37 represent distinct and potentially novel viral species, we performed capsid sequence alignments (Fig. S2) against their closest known relatives, SaIV1 and WIV1, identified via BLASTx (Fig. 2, Table S5). Contig 32 and contig 33 exhibited less than 90% sequence identity with their closest relatives (Table 1), falling below the ICTV species demarcation threshold for this virus family (https://ictv.global/report/chapter/iflaviridae/iflaviridae). These findings support their classification as novel members of the *Iflaviridae* family. Meanwhile, contig 37 shares 96.8% capsid sequence identity with the unclassified iflavirus sequence informally referred to as “watermelon iflavirus 1” (NCBI accession PQ058696.1), meeting the ICTV species threshold (≥ 90%) and indicating conspecificity (Table 1).

**Table 1:** Pairwise amino acid identity (%) of capsid proteins between three aphid iflaviruses represented by Contigs 32, 33, and 37 and their closest known relatives. SaIV1, Sitobion avenae iflavirus 1 (NCBI accession XIF87525.1); WIV1, watermelon iflavirus 1 (NCBI accession XDG29873.1). Contig 37 shares 96.8% capsid amino acid identity with WIV1, exceeding the ICTV species demarcation threshold for *Iflaviridae* (≥ 90%), consistent with conspecificity

Virus	Contig 32	Contig 33	Contig 37	SaIV1	WIV1
Contig 32	100				
Contig 33	67.4	100			
Contig 37	31.1	31.7	100		
SaIV1	59.3	58	32.2	100	
WIV1	31.4	31.7	96.8	32.5	100

### Host-association analysis of the iflaviruses

We initially presumed that *A. gossypii* was the host species for the three viruses represented by contigs 32, 33, and 37. However, because additional aphid species were detected in the Lee and Macon RNA samples (Table S4), it remained possible that these viruses are not actually associated with *A. gossypii*. To assess their aphid host association, we attempted to recover the 658-bp COI barcode sequences used for species identification from the individual aphid RNA samples that test positive for these viruses. PCR assays using virus-specific primers were performed on aphid samples collected from five counties in Alabama. Initial screening of the county-level pooled RNA samples detected contig 32 in Macon, contig 33 in Escambia, and contig 37 in both Macon and Lee counties. Based on these results, 30 individual aphid samples (each containing varying numbers of alates) from each virus-positive county were subsequently tested.

Individual PCR assays showed that one sample from Macon (containing one alate) was positive for contig 32, one sample from Escambia (six alates) was positive for contig 33, and two samples were positive for contig 37: one from Macon (one alate) and one from Lee (four alates) (Table 2).

**Table 2:** PCR assay results for three iflaviruses detected in aphid samples collected from five Alabama counties. The three viruses correspond to contigs 32, 33, and 37, respectively. For each virus and county, results are presented as the number of positive samples out of 30 tested. Numbers in parentheses indicate the number of alate individuals within each virus-positive aphid sample. “-“ denotes a pool-negative result, for which no downstream individual tests were conducted

County	Autauga	Escambia	Henry	Macon	Lee
Contig 32	-	-	-	1 (1)	-
Contig 33	-	1 (6)	-	-	-
Contig 37	-	-	-	1 (1)	1 (4)

These four RNA samples were used to synthesize cDNA and amplify the 658-bp COI barcode fragments, which were then cloned and Sanger-sequenced. The resulting barcode sequences were submitted to the BOLD Identification System as described above. The results showed that the Macon sample containing one aphid positive for contig 32 was derived from *R. rufiabdominale*, whereas the other three samples (one additional Macon sample, one Lee sample, and one Escambia sample; Table 2) were derived from *A. gossypii*. These findings indicate that the contig 32 iflavirus is associated with *R. rufiabdominale* iflavirus, while the contig 33 and 37 iflaviruses are associated with *A. gossypii*. Therefore, we propose the common names Rhopalosiphum rufiabdominale iflavirus (RrIV) for contig 32, Aphis gossypii iflavirus 1 (AgIV1) for contig 33, and Aphis gossypii iflavirus 2 (AgIV2) for contig 37.

### Phylogeny of aphid iflaviruses

**Fig. 4:**
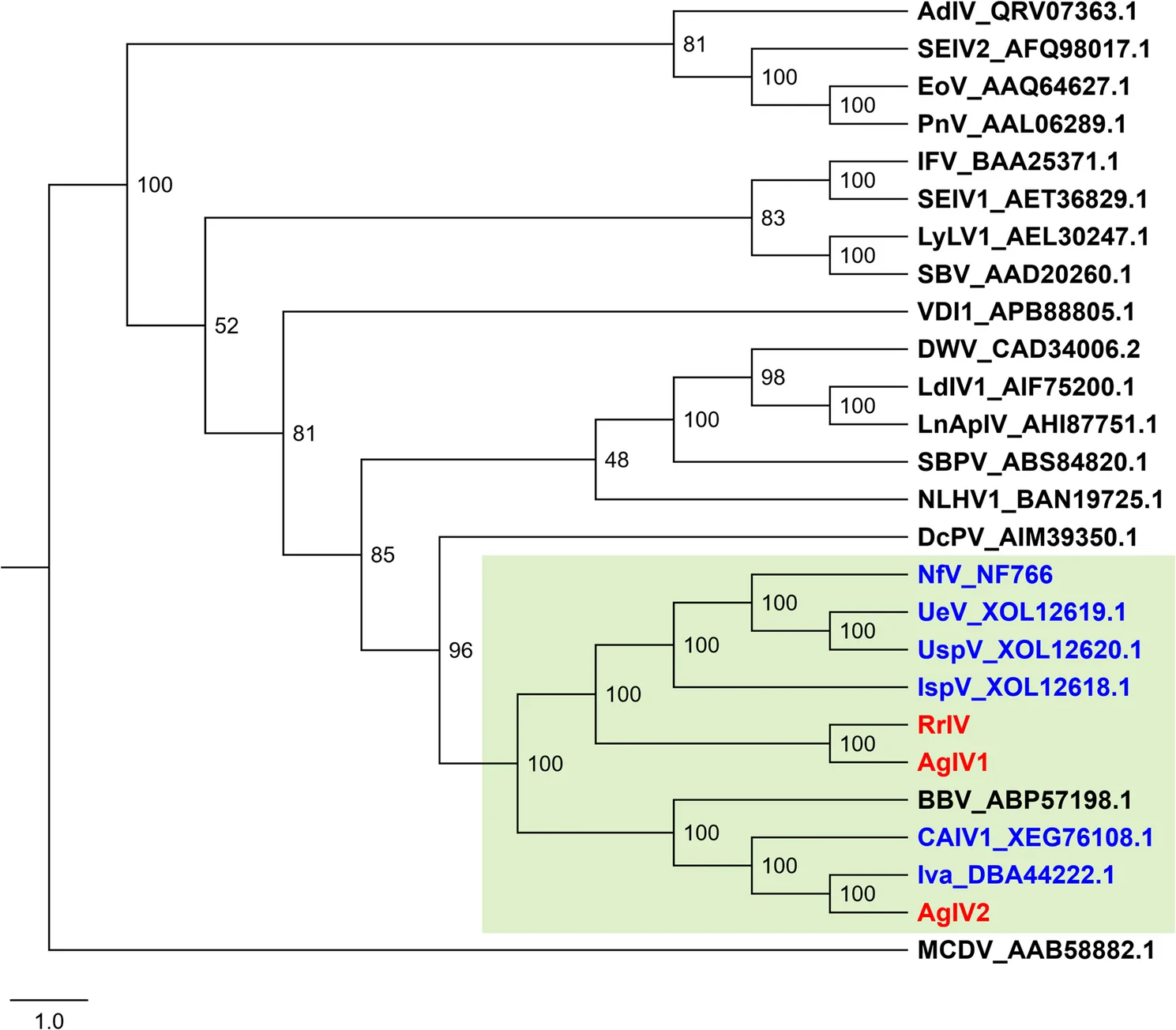
Bayesian phylogeny of aphid iflaviruses. Bayesian tree inferred from polyprotein amino acid sequences. ICTV-reconciled species are shown in black and labelled with species acronyms followed by NCBI accessions (see Table S7). Recently reported aphid iflaviruses from Rozo-Lopez et al. (2025) are in blue, using the same labelling convention. Viral sequences generated in this study (RrIV, AgIV1, and AgIV2) are highlighted in red. The tree is rooted with maize chlorotic dwarf virus (MCDV, family *Secoviridae*). Posterior probabilities are shown at nodes. A green box highlights the aphid-associated clade. Scale bar indicates expected substitutions per site

To elucidate the evolutionary relationships among these aphid iflaviruses, we conducted a phylogenetic analysis using their polyprotein sequences (Table S7). The resulting tree, rooted with maize chlorotic dwarf virus (MCDV; family *Secoviridae*) as the outgroup, confirmed RrIV, AgIV1, and AgIV2 as members of the *Iflaviridae* family. These viruses clustered with the ICTV-reconciled iflavirus species and six recently reported aphid-associated iflaviruses, forming a well-supported clade (posterior probability = 100%; Fig. 4).

All ten aphid-derived iflaviruses, including six species reported by Rozo-Lopez et al. [34], three identified in this study, and the ICTV-reconciled Brevicoryne brassicae virus (BBV), formed a distinct aphid-specific clade. This clade was strongly supported (posterior probability = 100%) and clearly separated from other *Iflaviridae* taxa associated with non-aphid hosts. Phylogenetic positioning suggests this aphid-specific lineage represents a recent evolutionary diversification within the *Iflaviridae* family (Fig. 4).

Further examination of the tree revealed two primary subclades within the aphid-specific clade (Fig. 4). Interestingly, despite being detected in the same host species, AgIV1 and AgIV2 did not cluster together. RrIV and AgIV1 formed sister lineages and grouped with four other aphid iflaviruses, whereas AgIV2 formed a separate subclade alongside BBV, Cavariella aegopodii iflavirus 1 (CAIV1), and *Iflavirus aphis* (Iva) (Fig. 4). Among these three new iflaviruses identified in this study, RrIV and AgIV1 exhibited the closest evolutionary relationship, whereas AgIV2 was more distantly related.

### Genomic characterization and negative-strand RNA detection of aphid iflaviruses

To validate and further characterize the genomes of the three aphid iflaviruses, each virus was re-sequenced via PCR amplification from county-level pooled RNA samples selected based on relatively higher viral abundance (Fig. 2), followed by DNA cloning and Sanger sequencing. Specifically, RrIV and AgIV2 genomic fragments were amplified from pooled Macon County RNA, whereas AgIV1 fragments were amplified from the Escambia pooled RNA. Complete terminal sequences at both the 5’ and 3’ ends were also determined, resulting in full-length genome assemblies. Alignments between PCR-derived sequences and RNA-seq contigs showed identical sequences across overlapping regions, confirming assembly accuracy. Contigs 32, 33, and 37 covered 99.4%, 99.2%, and 96.1% of their respective full-length genomes.

**Fig. 5:**
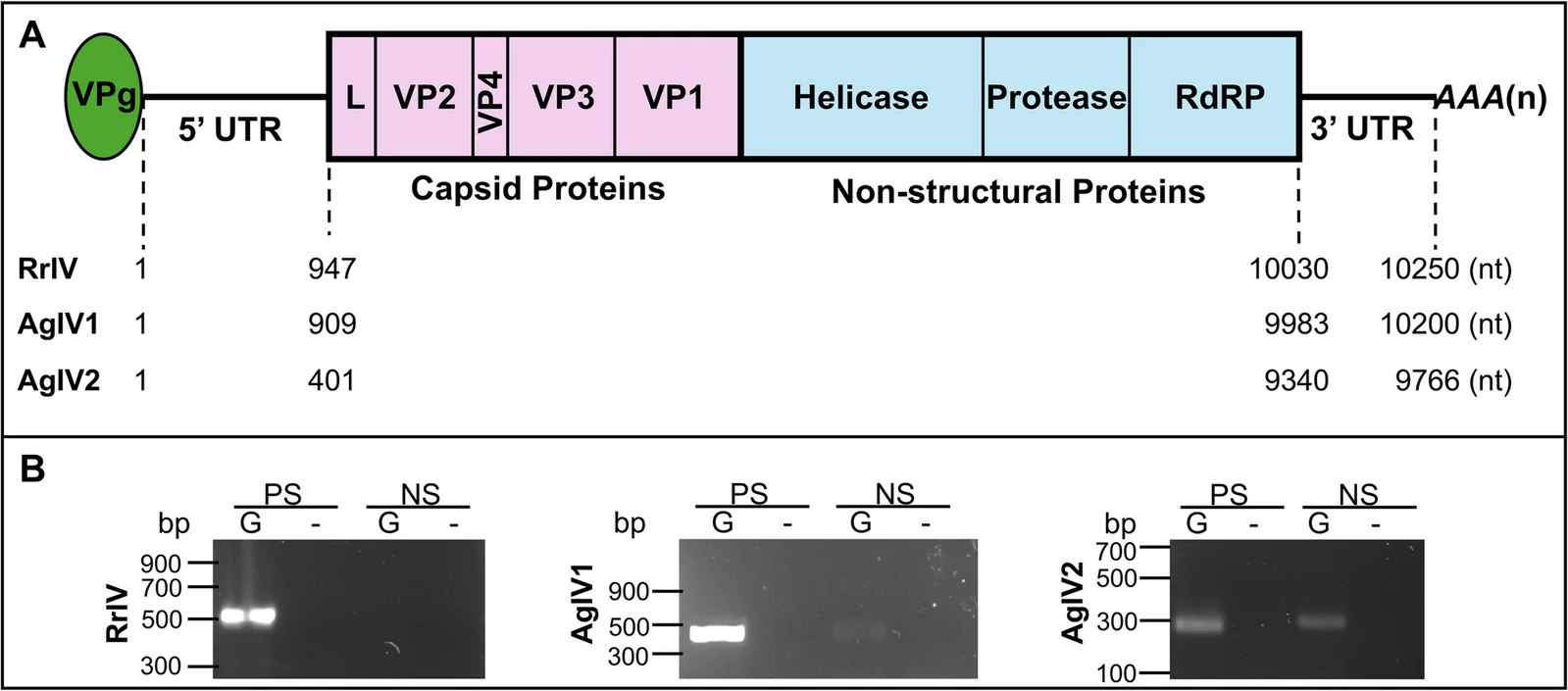
Genome organization and replication evidence of three aphid iflaviruses.** A** Predicted genome architecture of RrIV, AgIV1, and AgIV2 (schematic, not to scale). Each genome contains a single open reading frame (ORF) encoding a polyprotein comprising structural (capsid) and non-structural proteins. **B** Strand-specific RT-PCR targeting the RNA genomes. Lanes show PCR product from reactions specific for the positive-strand (PS) and the negative-strand (NS) RNA genomes (G). No-template controls (-) are included for each primer set. Detection of NS products for AgIV1 and AgIV2 provides evidence consistent with replication in host cells, whereas RrIV showed no NS signal under these conditions

Sequence analysis revealed that the full-length genomes of RrIV, AgIV1, and AgIV2 are 10,250 nt, 10,200 nt, and 9,766 nt, respectively, excluding the 3′ polyadenylated tails (Fig. 5A). Each genome contains a single open reading frame (ORF) encoding a polyprotein that encompasses both capsid and non-structural proteins. ORF lengths were comparable across the three viruses: 9,084 nt for RrIV, 9,075 nt for AgIV1, and 8,940 nt for AgIV2.

In contrast, the untranslated regions (UTR) at both termini exhibited greater variability. The 5’-UTRs measured 946 nt, 908 nt, and 400 nt, while the 3’-UTRs spanned 220 nt, 217 nt, and 426 nt for RrIV, AgIV1, and AgIV2, respectively (Fig. 5A). These differences may reflect lineage-specific regulatory elements or replication dynamics.

Strand-specific PCR analysis detected negative-strand RNA in AgIV1 and AgIV2, but not in RrIV, suggesting that the two *A. gossypii* iflaviruses actively replicate within their aphid host (Fig. 5B).

## Discussion

We performed metatranscriptomic analysis of aphids collected from cotton fields in five Alabama counties. Because *A. gossypii* is the dominant aphid species in Alabama cotton fields during our sampling period (late June to early July) [12], we expected that most collected specimens would be *A. gossypii*. However, not every individual aphid was morphologically identified prior to pooling. COI sequence analysis showed that only *A. gossypii* was detected in three counties (Autauga, Escambia, and Henry), whereas two counties (Lee and Macon) contained additional aphid species.

Across the dataset, we detected seven viral families by RNA-seq, six of which were validated by PCR and Sanger sequencing. These include three insect-associated families—*Dicistroviridae*, *Iflaviridae*, and *Nudiviridae*. Members of *Dicistroviridae* include key model systems. For example, cricket paralysis virus has been pivotal for studies of IRES-mediated (internal ribosome entry site-mediated), cap-independent translation—where viral RNA recruits ribosomes directly without a 5’ cap or scanning—and host-virus interactions [37]. This family also contains economically important pathogens, such as acute bee paralysis virus, which affects managed pollinators [38]. Unlike *Dicistroviridae* [39] and *Iflaviridae* [40], which are positive-sense, single-stranded RNA viruses, *Nudiviridae* are double-stranded DNA viruses reported from multiple insect orders, including Coleoptera, Diptera, Lepidoptera, and Orthoptera [41]. Although active nudivirus infections have not been confirmed in Hemiptera, we detected nudivirus-like transcripts in aphids by RNA-seq (five contigs > 1 kb) and by RT-PCR using DNase-treated RNA, minimizing the possibility of genomic DNA carryover. The recovery of multi-kb nudivirus transcripts is consistent with active transcription in aphids, though expression from endogenous nudivirus elements cannot be excluded without minus-reverse transcriptase controls and genomic assays. If subsequent tests confirm productive infection, the host range of *Nudiviridae* would extend to Hemiptera.

*Mitoviridae—*formerly placed within *Narnaviridae*—are capsidless, positive-sense single-stranded RNA viruses with extremely simple genomes that typically encode only an RNA-dependent RNA polymerase (RdRp) [42]. Mitoviruses replicate in mitochondria and were first characterized from fungi, with plant-associated lineages reported more recently [43, 44]. Reports of mitovirus-like sequences in animals, including insects, are emerging [45, 46], although host assignment can be challenging in metatranscriptomic datasets. Given the presence of mitoviruses in cotton viromes [33], it remains uncertain whether mitovirus-like reads in our aphid libraries derive from aphids, their host plants, or associated fungi. By contrast, CLRDV (*Solemoviridae*) detected here is unequivocally a plant-infecting virus and a known *A. gossypii*-transmitted pathogen. Its frequent detection across southern U.S. cotton fields makes its occurrence in the *A. gossypii* non-host read fraction expected [47].

Variation in relative viral abundance was observed among county-level libraries in the RNA-seq data. However, abundance of RNA-seq reads depends on both viral load and the number of infected individuals, and therefore does not directly reflect how widely a virus is distributed in the population or across counties. These patterns should thus be interpreted descriptive only, as they are based on normalized and scaled read counts from pooled samples and do not reflect absolute viral abundance or infection frequency.

We also recovered two Partitivirus-like contigs which grouped with *Betapartitivirus* and *Deltapartitivirus*, respectively, in phylogenetic analysis (Fig. S1). Betapartitiviruses infect plants and fungi, whereas deltapartitiviruses are plant-infecting [48]. Their detection in field-collected aphids—particularly the deltapartitivirus contig—is most parsimoniously explained by plant-mediated acquisition and does not, by itself, establish replication in insects. Although this raises the question of whether *A. gossypii* could transmit certain partitiviruses, aphid transmission of partitiviruses has not, to our knowledge, been demonstrated. Notably, these contigs differ from sequences previously reported from cotton viromes, which clustered within *Gammapartitivirus*, a genus considered fungal-restricted [29, 33, 48].

Three iflaviruses (RrIV, AgIV1, and AgIV2) were identified from the aphid virome. PCR analysis of individual RNA samples indicated that two different Macon samples (each containing a single alate) tested positive for RrIV and AgIV2, respectively, and one Escambia sample tested positive for AgIV1 (Table 2). Because the Escambia samples contained only *A. gossypii*, based on COI sequences retrieved from the RNA-seq assembly, *A. gossypii* was expected to be the host of AgIV1. This was confirmed by individual PCR testing, which showed that the COI sequence amplified from the AgIV1-positive RNA sample matched *A. gossypii* as its top hit.

Because RrIV and AgIV2 were detected in the Macon samples, which contained aphid species in addition to *A. gossypii*, further assays were required to determine their actual hosts. PCR assays of the single-alate Macon samples that tested positive for these viruses supported association of RrIV with *R. rufiabdominale* and AgIV2 with *A. gossypii*.

Phylogenetic analysis indicates that RrIV, AgIV1, and AgIV2 cluster with other aphid-associated iflaviruses, together forming an aphid-specific clade within *Iflaviridae* (Fig. 4). Despite being associated with the same host species, AgIV1 and AgIV2 do not cluster together, suggesting complex evolutionary histories—a pattern consistent with previously proposed cross-species horizontal transmission of aphid iflaviruses [34]. RrIV and AgIV1 form sister lineages within one subclade, whereas AgIV2 groups with the alternate subclade that includes BBV (Fig. 4). This phylogenetic pattern mirrors their genome architectures: RrIV and AgIV1 share similar ORF lengths (9,084 nt; 9,075 nt), 5′-UTRs (946 nt; 908 nt), and 3′-UTRs (220 nt; 217 nt); by contrast, AgIV2 possesses a shorter ORF (8,940 nt) and distinct UTR lengths (5′-UTR: 400 nt; 3′-UTR: 426 nt).

In contrast with a recent report that some aphid iflaviruses can reach 100% prevalence in field populations [34], all three newly identified aphid iflaviruses exhibited low prevalence in our samples, with only one sample positive for RrIV, one for AgIV1, and two for AgIV2. Accordingly, we propose the names *Iflavirus furtiva* (RrIV), *Iflavirus obscurata* (AgIV1), and *Iflavirus rarivira* (AgIV2). While *I. furtiva* and *I. obscurata* represent newly identified viral species, *I. rarivira* comprises AgIV2 together with a previously deposited sequence, “watermelon iflavirus 1” (WIV1), from watermelon (*Citrullus lanatus*), a common host of *A. gossypii*. Given the species-level identity between AgIV2 and WIV1, plant-mediated transmission to aphids is plausible. Alternatively, the watermelon library may have contained *A. gossypii* tissue infected with AgIV2.

Iflaviruses can act as pathogens detrimental to their insect hosts [49, 50]. However, recent work in aphids support a symbiotic association: aphid iflaviruses can be vertically transmitted, show no detectable fitness costs, and localize to multiple tissues, including bacteriocytes—specialized insect cells that house intracellular bacterial symbionts [34]. Although the three aphid iflaviruses identified in the present study are closely related to these previously characterized aphid-associated iflaviruses, their effects on their aphid hosts remain unknown. Future work should determine whether they behave as neutral, beneficial, or pathogenic associates and clarify their consequences for aphid physiology and ecology.

## Conclusions

Metatranscriptomic profiling of field-collected aphids across five Alabama counties reveals a taxonomically diverse virome with county-level differences in relative viral RNA-seq read abundance. In addition to numerous viruses lacking confident family-level assignments, seven viral families were detected by RNA-seq, six of which were PCR-validated, spanning both insect-infecting and plant-pathogenic taxa. We identified three iflaviruses, assembled their complete genomes, and evaluated replication-associated signals. Together, these results establish a genetic baseline for dissecting aphid –virus interactions and evaluating their effects on cotton production systems. 

## Supplementary Information

Supplementary Material 1: Fig. S1. Bayesian phylogeny of partitiviruses detected in cotton field-collected aphids.

Supplementary Material 2: Fig. S2. MAFFT alignment of capsid protein sequences from three aphid iflaviruses and two reference viruses, Sitobion avenae iflavirus 1 (SaIV1) and watermelon iflavirus 1 (WIV1).

Supplementary Material 3: Table S1. Field collection of aphid alates in Alabama, summer 2024.

Supplementary Material 4: Table S2. Primers used in this study.

Supplementary Material 5: Table S3. Statistical summary of RNA sequencing for aphid alates collected in five Alabama counties.

Supplementary Material 6: Table S4. COI-based identification of aphid species present in county-level RNA samples.

Supplementary Material 7: Table S5. Viral contig annotation from the aphid transcriptome.

Supplementary Material 8: Table S6. Partitiviridae viral species used in phylogenetic analysis.

Supplementary Material 9: Table S7. Iflavirus species used in phylogenetic analysis.

## Data Availability

The sequence reads and the full-length RrIV, AgIV1, and AgIV2 genomes generated and/or analyzed in this study are available in the NCBI database under accession number PRJNA1363914. The putative viral contig sequences in FASTA format are publicly accessible in the GitHub repository: https://github.com/chaoyang-zhao/Aphis-gossypii-virome.

## Abbreviations

RrIV: Rhopalosiphum rufiabdominale iflavirus
AgIV1: Aphis gossypii iflavirus 1
AgIV2: Aphis gossypii iflavirus 2
ALPV: Aphid lethal paralysis virus
BBV: Brevicoryne brassicae virus
CLRDV: Cotton leafroll dwarf virus
ICTV: International Committee on Taxonomy of Viruses
RACE: Rapid amplification of cDNA ends
RdRp: RNA-dependent RNA polymerase
RPKM: Reads per kilobase per million total reads
SaIV1: Sitobion avenae iflavirus 1
WIV1: Watermelon iflavirus 1
UTR: Untranslated region
ORF: Open reading frame
