# Supplementary material for: Novel iflavirus species discovered through virome analysis of cotton field-collected aphids
Source: BMC Genomics. 2026 Jul 10;27:809. doi: 10.1186/s12864-026-13132-9 (PMC13637338; doi:10.1186/s12864-026-13132-9)
Supplement: Supplementary file 1 [file 12864_2026_13132_MOESM2_ESM.pdf]

Fig. S2. MAFFT alignment of capsid protein sequences from three aphid iflaviruses and two reference viruses, *Sitobion avenae* iflavirus 1 (SaIV1) and watermelon iflavirus 1(WIV1). Three aphid iflaviruses identified in this study are represented by Contigs 32, 33, and 37, respectively.

```
           1 ........10........20........30........40........50........60
SaIV1      1 MTFENKFNI-----------------AFSGVARDPTYAKPEYYSDIKDGILYRFDKNDWD
Con32      1 MMSFNEHNI-----------------AITGAPRCSTYAPAKYFDVIQGDILYRFDENDWD
Con33      1 MIV----PT-----------------IIVSAPFVSDSGVTCIID----GVLKRFCEKTWE
Con37      1 MSSQNSASVYQIYATSSSDVATSSDESPIGSTNVGTFRSVDHLTVSFSRCCDKIEKYYQR
WIV1       1 MSSSNSASVYQTYATSSSDVATSSDDSTIGSTNIGTFKSVDHLTVSFSRCCDKIEKYYQR

          61 ........70........80........90.......100.......110.......120
SaIV1     44 YCQVISLDVVTPGFSYAHACGDSKLPQILIVR----------PRPIPVRNAPLRPSDAIE
Con32     44 YCQIISLKKVVQGFSYAHACGDSQLPQFRIVRE--------QSRPIPVRDAPLRPSDAVE
Con33     36 WINVCSLNEFVGQQSFAQRVGDSGLPQMSVRYV--------YSRPIPVRDAPLRPSDAIE
Con37     61 RNNLVSRIKTLRASSQAKLIPPPNVPQFEVEKEPLCFERKLVDSRLYSEVAETPSSSSSE
WIV1      61 RNNLVSRIKSLRASSQAKLIPPPNVPQFEVEKEPLCFERKLTDSRLYSEVAELPASSSSE

         121 .......130.......140.......150.......160.......170.......180
SaIV1     94 AACYRRAQAMTRSRRVYKFSYGQQRLFINGIPCQGPLTRSQARKKRVFSLRKIEERRVAA
Con32     96 AACYRRAQALLRGKSTYRFSYGQQRLFINGIPCQGPLTRSQAKKKRAFSLIKIAERREAH
Con33     88 AACYRRAQALTRSKRVYKFSYGQQRLFINGIPCQGPLTRSQAKKKRTFALNKIFERRAAA
Con37    121 EVLFNVPIVRT---NIYLINNLQKQININ----EWRLNKQINKRKRLDQLMK---NQVAY
WIV1     121 EEIFNVPIVRT---NLYLINNLQKQININ----EWRLNKQINKRKRLDQLMK---NQVAY

         181 .......190.......200.......210.......220.......230.......240
SaIV1    154 KEQLRLERFERLWGEGARTFLQTLGYKKRFIAPYVPFVSLPTSIRQLKRTTPRSSINCSV
Con32    156 KEQLRLERFEKLWGPGVRTFLRSLGYKKVHIEPYVPFPALPTSVKQIKNTTSRSSIDCSE
Con33    148 KEQLRLERFERLWGKSNYSFLKSLGYKKVHIAPYVPFPALPTSVKQVKHVIARSAIHCSE
Con37    171 SPVF------------NPSPVYNPTTEPINQTEYRPFPAMPTSTKSKKTRKSRGSIHDSI
WIV1     171 SPVF------------NPNPVYNPTTEPISEQEYRPFPALPTSTKTKKCRKSRSSIHDSI

         241 .......250.......260.......270.......280.......290.......300
SaIV1    214 LGTFVPIIEAVRDSGLAFKLRQRIKGFRTAFVKVQCKSVSLRDCIRATHVMSCTPQMDQG
Con32    216 KGTFVSIPSAIRDSGLAQKLRQLIKSFRVQFVHLPTRCVSLREAIRKVYVPIVCCQMDAG
Con33    208 LGTFMARPVAVRDLQLAYKLRQIIKNFRPNYRSIPTRSVSLRDVIRKVFIPIVKCQMDAG
Con37    219 FGVYVPKYTATRDINLAFKLRLKIRSFRHTITNHYINYLLYYKF--KILTYPIYPQMDA-
WIV1     219 FGVYVPKYTATRDVNLAFKLRLKIRSFRYTITNHYINYLLYYKF--KILTYPIYPQMDA-

         301 .......310.......Con320.......330.......340.......350.......360
SaIV1    274 SHGVPDDITAERISNTVITTHNEESVGASPTTVL-NWHSLTTTDPEQEYNFGTDRWNIYD
Con32    276 PHGSDDQVTNEKISNTVVTTHSEESRGESDVTVL-NWHSLTTTEQEQEYTFGTERWSIYD
Con33    268 PHGSDDTVTSEKISNTVVTTHSEYSTGASDSTVL-NWHTLTTTEPEQEYTFGTERWSIYD
Con37    276 -HGEEGSVSADKKLNTVIATQRDDSVGVSTPIIYPEWQELVTSDHFSSYNTSTNRWLKYK
WIV1     276 -HGEEGSVSADKKLNTVIATQRDDSQGVSTPIIYPEWQELVTSDHFSSYNTSTNRWLKYK

         361 .......370.......380.......390.......400.......410.......420
SaIV1    333 VITWDKNQVSGSGVD-------KISIPVDLMAKCWSQPSMALFKSNRYWRSDIELRFQLN
Con32    335 VIDWTGDTFICPDTDHNKENRCSFDIPSSLFKKCWSQPAMAIFKNNRYWRSDIELRFQLN
Con33    Con327 VIDWSGSSFVCPGSS-IKEHTCGFNIPADIMKKCWTQPSMAIFKNNRYWRSDIELRFQLN
Con37    335 TLAWSNNKIT--PID-------KINVPSDLLTELVNTPLFIPFRNNRYWKGDFEFKFVLN
WIV1     335 TLAWSNNKIT--PVD-------KINVPSDLLTELVNTPLFIPFRNNRYWKGDFEFKFVLN

         421 .......430.......440.......450.......460.......470.......480
SaIV1    386 TNKFQTGSLQCSYIYDCADDGG-LSYRGNVYCLSHTNHCILDASGSNMGTLLIPYKFNNP
Con32    395 TNKFQTGSVMIGYIYNTTNDNS-LNYRLNINNFSQTNHCIIDASGSNLGTLMIPYKFNNP
Con33    386 TNKFQTGSVMIGYMYNTSNDSS-QKFRVNLANLSQTNHCIIDASGSNLGTLYIPYKHNNP
Con37    386 SNKFQQGSLQISFMYCSDLEQSFNTYRNNIYCGSQTNHCILNAGSSNEGTLIIPFKYFNP
WIV1     386 SNKFQQGSLQISFMYCSDLEQSFNTYRNNIYCGSQTNHCILNAGSSNEGTLIIPFKYFNP

         481 .......490.......500.......510.......520.......530.......540
SaIV1    445 YINTDNDKE--YCRVFIKVLNALDCPSTVMGSASLTVYYRFINAKFMGTLDNALIVPPKP
Con32    454 YINTDNDYN--LCTVYMLVLNELDSPSTVYNHASITIYYRFVNAKFMGTLDTSLVV--KP
Con33    445 YLNTDNFHN--MCRINLIILNDLDSPSTVYNHASITIYYRFVNAKFMGTLDTSLVI--KP
Con37    446 VITTNPKENVDYCKIFIRTLNPLVCSSTTFNSADLTIFINLNNAYFTGSIDFTLIQ--QP
```

```
WIV1    446 VITTNPKENVDYCKIFIRTLNPLVCSSTTFNSADLTIFINLNNAYFTGSIDFTLIQ--QP

        541 .......550.......560.......570.......580.......590.......600
SaIV1   503 QMLTLMRALGAAAEAGLRSVNKDLNRDKPPEPSCPVPFTPRSTSSWCIGTNLVEPTNLLR
Con32   510 QMLSLMRILGAAAEAGVRQANKDLNRDKPPEPSPPVPMTPRSSSSWCAGTNIVEPVNLLR
Con33   501 QMLTLMRALGAAAEAGLRQVNKDLNRDKPPEPSAPVPFAPRSTSSWCVGTNLVEPTNLLR
Con37   504 QMFHLMRALIKTTEFGLNQIDPDSNRDNPPDISTPTSVTITNASSWCTGTNSIEPVNHLR
WIV1    504 QMFHLMRALIKTTEFGLNQIDPDSNRDNPPDISTPTNITITNASSWCTGTNSIEPVNHLR

        601 .......610.......620.......630.......640.......650.......660
SaIV1   563 LDSTGQTPHPNLLLSSMKVKDIVNIFGIIVLDLKWSVDDKVGANLLRLDGAPIQKFSDGP
Con32   570 LDNSGQTPHPNIKLSDMHVKSIVSKYGLIKRSIKWKTDYSSGKILYTFDAAPVFKYSEYN
Con33   561 LDNSGQTPHPNLRLSDMHVNSIVSIYGLIYSNLKWQTDHVSGHVLYKMDAAPVFKYSEYS
Con37   564 LDTLGQTPHPNLQHDELKTSVITRIFGLI-HTVEWKFSHAAGDHLLTIEAAPMSNLLTYP
WIV1    564 LDTLGQTPHPNLQHDELKTSVITRIFGLI-HTVEWKFSHAAGDHLLTIEAAPMSNLLTYP

        661 .......670.......680.......690.......700.......710.......720
SaIV1   623 SAPINWPIYPSADTAYPLPPISFISSLFSYWRGTMDLRIDIVGTPFHKGQLLISYIPRNH
Con32   630 KCSINWPVYPHAEEMYGIPPVAVISSLFGYWRGTLDLKIDIVGTPFHKGQLLVSYIPHNK
Con33   621 SIPVESDTYPHAESMYCIPPISVISALFGYWRGTLDLKIDIVGTPFHKGQLLVSYVPHNH
Con37   623 SQVVT--LKNKTYTLYNLPPVAVISNLFAYWRGTLEIRIDFVATSFHTGSLLIAYIP---
WIV1    623 SQVVT--LKNKTYTLYNLPPVAVISNLFAYWRGTLEIRIDFVATSFHTGSLLIAYIP---

        721 .......730.......740.......750.......760.......770.......780
SaIV1   683 QLDEVSNKVTMEMAKSSNYTICDLSETRSFTINIPYISSRPMYPVKYSQG----RGSDIP
Con32   690 QMDKLRNDVTIDIAKSSSYTICDLSETRSFSINIPYISNRHMYPVRNTSL----DTH--P
Con33   681 QMDILKNNITIDIAKSSNYTICDLSETRSFSINIPYISSKHMYPVRGSVG----NSHSIT
Con37   678 ---GIKNTVTIDQARSSNYVICSLQEQQTYTIRVPYIANSPSWPRKYNKSYKIHNSSEIN
WIV1    678 ---GIKNTVTIDQARSSNYVICSLQEQQTYTIRVPYIANSPSWPRKYNKSYKIHNSSEIN

        781 .......790.......800.......810.......820.......830.......840
SaIV1   739 PPGYITVFVLNPLIPMDQVSRTVSINMYLKAGSDFELLVPCQPSIGLIYDPVYKPEPNNV
Con32   744 PPGFVNIFVLNPLVPMDNVTPYVYLNVYLRGGQDFELLVPIQPSVGLPYDPSYKPEVENI
Con33   737 PPGYVNIFVLNPLIPMDNVTSYVYLNIYLRAGKDFELMIPVQPSIGLPYDPSFKPEEKNL
Con37   735 PPGSIQFFVLNPLIPLDNVYSSINININYYIRGGTDFEVLVPIQPNISTPYNLDYKDNKFNE
WIV1    735 PPGSIQFFVLNPLIPLDNVYSSINININYYIRGGTDFEVLVPIQPNISTPYNLDYKDNKFNE

        841 .......850.......860.......870.......880.......890.......900
SaIV1   799 VKVNRPINPPNKAGLIPYI----------------YGTTQGILYVSSNLNAYMGNLVTRNF
Con32   804 IYT---QETTGKCCLLPY-----------------YTKQSPLLFLSHNLVLYKNQIITSSD
Con33   797 IKTTLSENPSNKATLLPYL----------------YSTSQPIIYLSTNLNTYNNRIIPLTT
Con37   795 MPL---TSASGTSTVFNYLISSPTGLPNSYVTLVYAPTDRLTPISYE-ELRDGQIISKNN
WIV1    795 MPL---TSASGTSTVFNYLISSPTGLSNSYVTLVYAPTDRLTPISYE-ELRDGQIISKNN

        901 .......910.......920.......930.......940.......950.......960
SaIV1   844 -ISDFVVQILDTPFIK-IEKYTIRLISPIK-SNILYDRIARTWLPTITFN------YAAI
Con32   845 -ASIFVVKLKEGPPK--DPNFILRLPTSFS-TILIKDR------KVVTFN------YGAM
Con33   842 -LPVHLITFPTKPFEGDEPNFVLRFSCSLH-CCLIKDR------PDVVFS------YGAL
Con37   851 RYTDLFDLIPDQPGI--LP-FYLESSMPVNFSITTPDK------QNVFYSCSIFVLYNEL
WIV1    851 RYTDLFDLIPDQPGI--LP-FYLESSMPITFSITTPDK------QNVFYSCSTFVLYNEL

        961 .......970.......980.......990......1000......1010......1020
SaIV1   895 CEFSPGKFGLVPIEKEFIKWYSIEMKKP-FTGLVLESEWVAYYTV-DATDENRNKFCKDV
Con32   889 LLILDNVYAIAPIEKAFIEAYNYKNKPKLFTGVITADEWISYYTV--VNDTGKNTEQLIY
Con33   888 CLRAVDSYSIVPIEKAFVDNYESKYKKK-FTGLVTKQEWVDYYNIPDGDDLGKNIRQSNF
Con37   902 CRNEGHTILWPVISKEAAQKYAR-----------SDQSWSQL--VPALIPALKCYKNSDT
WIV1    902 CHNEGHTILWPVISREAAQKYAR-----------SDQSWSQL--VPALIPALKCYKNSDT

       1021 ......1030......1040......1050......1060......1070......1080
SaIV1   953 YNLFDDYVLKVPKATVQCPFWL--HSDPQMDERHLSVNSVVPMSGSLISTSSGLTILGES
Con32   947 IKPFHDFVMKVPNNLYNSFEWR--LVDPQMEEREIDSNKIIPMSGRLQSTNSGVLTLGED
Con33   947 RVPYFNYVMKVSHTTYTSPDWV--WLEPQMDERIVDSNRIIPMSGSLQPTGSGLLTLGEN
Con37   949 VNI--TFLTKIFVHVHNVELLLDDYCFPQNEER-LNSNNFLDMTQSLPTTGYGLQLYGEN
WIV1    949 VNI--TFLTKIFVHVHNVDHLLDDYCFPQNEER-LNSTNFLDMTQSLPTTGYGLQLYGES
```

```
 1081 ......1090......1100......1110......1120......1130......1140
SaIV1 1011 FSDLKTLCRRYQKLFNANIILDNTRTDKMSIC-FPVTPLGLINNSKNVDSTIPELFNRCR
Con32 1005 FSDLKTLCRRYQKLFNTTILLDNKHTDIMSVR-FAVSPLGIINNSDGATQNIPEIFNRCR
Con33 1005 FSDLKTLCRRYQKLFNIRIILDNLHTDKMSVR-FAASPLGIINNSVSEDNTIPEVFNRCR
Con37 1006 FNDLKNYCRRYQYYTNISYKNDNPLIKKQALAIIPLVPQGLVNSLIYNDNTVNHIVNRCK
WIV1  1006 FNDLKNYCRRYQYYTNISYKNDNPLIKKQALAIIPLVPQGLVNSLIYNDNTVNHIVNRCK

      1141 ......1150......1160......1170......1180......1190......1200
SaIV1 1070 DGVIPLIASGFRYWRGSMRYRITTDSRLDINIVVQHRPDVTSSMYEVTLITDNQDR-NSY
Con32 1064 DGIIPIIASGFRFWRGSIRYRIMTDSKYDLNIIVQHRPDVLTTVYDIIKITSTDKA-ESY
Con33 1064 DGIIPILASGFRFWRGSLRYRLMTDSKYDLNMIIQHRPDVTTSVYNIVLIDANDET-SSY
Con37 1066 DGFIPIISSGYRFYRGGLRLRIVSDTRRNINLWVQHRPEMLLKFLQTHIIDPDNQSLSDY
WIV1  1066 DGFIPIISSGYRFYRGGLRLRIVSDTRRNINLWVQHRPEMLLKFLQTHIIDPDNQSLSDY

      1201 ......1210......1220......1230......1240......1250......1260
SaIV1 1129 LNHGYSMVIQNLNVNNCVDIEVPWYSPYMLNSCHPGNNHDGKD-------LKFSLGSLYV
Con32 1123 LNHGYSMIIQNLNINNCVDIEVPWYNPNMLASCHPPVKEGFYN--------NYCLGSLFI
Con33 1123 LNHGYSMVIQNLNLNNCVEIEVPWYNQNMLASSHPPIVQTDFN--------TYCLGSIFV
Con37 1126 FNHSYSFTIQNLELNNVLSLEIPWYSKNMLGCSFLPKVQSSSSDINAQKQTAFSLGSLVL
WIV1  1126 FNHSYSFTIQNLELNNVLSIEIPWYSKNMLGCSFLPKVQSSSSDINAQKQTAFSLGSLVL

      1261 ......1270......1280......1290......1300......1310.
SaIV1 1182 GFNNSS-SLKQ-VGISVYYSIGDDFRFDTFQGFPMII---DVNHAAIASPQ
Con32 1175 GFNNNV-NINN-VGLSVYYSLGDDFRFDTFQGFPAVI---RTLKTAVAQPQ
Con33 1175 GFSNSK-SIGS-IGLSVYYAIGDDFRFDTFQGFPAMI---RTSTTAIAQPQ
Con37 1186 GIDASPLELKTPINFEIFYSLADDFHFNTFQGFPLMLTLDQIPSTYQAHPQ
WIV1  1186 GIDASPLELKTPINFEIFYSLADDFHFNTFQGFPLMLTLDQIPSTYQAQPQ
```
